# Ongoing transmission of human onchocerciasis in blackflies in the medical district of Mont Ngafula 1 in Kinshasa after nearly two decades of uninterrupted onchocerciasis mass campaigns using community-directed treatment with ivermectin strategy

**DOI:** 10.11604/pamj.2026.53.1.43126

**Published:** 2026-01-05

**Authors:** Jean Claude Makenga Bof, Paul Mansiangi, Josué Zanga, Félicien Ilunga-Ilunga, Ako Aime Gilles Adjami, Sanfo Moussa Sounkalo, Didier Bakajika, Yves Coppieters

**Affiliations:** 1 School of Public Health, *Université Libre de Bruxelles (ULB), Route de* Lennik 808, Brussels, Belgium,; 2 Université Notre-Dame du Kasayi, Kasaï Central, Kananga, Democratic Republic of Congo,; 3 School of Public Health, Faculty of Medicine, *Université de Kinshasa (UNIKIN)*, Lemba, Kinshasa, Democratic Republic of Congo,; 4 Institut Supérieur des Techniques Médicales (ISTM), Lemba, Kinshasa, Democratic Republic of Congo,; 5 Expanded Special Project for Elimination of Neglected Tropical Diseases (ESPEN), World Health Organization, Brazzaville, Republic of Congo

**Keywords:** Onchocerciasis, entomological study, O-150 PCR, blackfly, Democratic Republic of the Congo

## Abstract

**Introduction:**

the medical district of Mont Ngafula 1 in Kinshasa, the capital city of the Democratic Republic of Congo (DRC), has been treated for onchocerciasis for over 16 years using Community-Directed Treatment with Ivermectin strategy (CDTI). This study aimed to determine blackfly infectivity rate and annual transmission potential in the Mont Ngafula medical district using O-150 PCR assay after four additional annual rounds of mass drug administration (MDA) following the initial entomological survey from August 2014 to July 2015.

**Methods:**

this was a longitudinal entomological study. Blackflies were collected at the Kimwenza collection site in the Mont Ngafula medical district from 1^st^ August 2019 to 31^st^ July 2020 using the human landing collection technique. The WHO-recommended O-150 pool screening polymerase chain reaction assay was used to determine the blackfly infectivity rate and annual transmission potential.

**Results:**

a cumulative 3,875 female blackflies were collected, and Simulium squamosum was identified as the main vector species. The infectivity rate was 0.75 % (95% CI: 0.48-1.13) with an annual transmission potential of 254 (95% CI: 153-396).

**Conclusion:**

these findings confirm the persistent transmission of human onchocerciasis in the Mont Ngafula 1 medical district despite 16 years of ivermectin distribution using the Community-Directed Treatment with Ivermectin strategy. They highlight the urgent need for alternative treatment strategies to accelerate the interruption and elimination of onchocerciasis transmission.

## Introduction

Human onchocerciasis, commonly known as 'River Blindness,' is a parasitic tropical disease caused by the filarial worm *Onchocerca volvulus (O.V)*, transmitted through repeated bites from infected blackflies of the genus Simulium [1,2]. This disease predominantly affects sub-Saharan Africa (SSA), spanning 28 countries, and extends to Brazil, Venezuela, and Yemen [3,4]. As of 2022, an estimated 246 million people required preventive chemotherapy for onchocerciasis, with 99% of them in SSA [3,4]. Nigeria and the Democratic Republic of Congo (DRC) rank as the highest endemic countries in the world, accounting for more than 42% of the global population requiring preventive chemotherapy [3,4]. In the DRC, all 27 provinces have been identified as endemic for human onchocerciasis based on Rapid Epidemiological Mapping of Onchocerciasis (REMO) surveys, supported by the Africa Program for Onchocerciasis Control (APOC) [5]. Three medical districts in Kinshasa- Ngaliema, Nsele, and Mont Ngafula 1 - were classified as meso-endemic, warranting the implementation of mass drug campaigns using the Community-Directed Treatment with Ivermectin (CDTI) strategy [5,6].

The first campaign in Kinshasa using the CDTI strategy was initiated in 2003. An initial entomological survey in the district of Mont Ngafula 1 was conducted between August 2014 and July 2015 to evaluate the impact of 16 years of CDTI on entomological indicators to inform future programmatic actions [5,6]. This initial survey collected 2,573 female flies from two sites (1,296 at S1 and 1,277 at S2) using the Human Landing Collection (HLC) technique and *Simulium squamosum* was identified as the main vector species [6]. Of the dissected flies, only 5.45% were parous, with 27% being infectious. The calculated annual biting rates at the first and second collection points were 5,269 and 5,183 respectively [6]. The O-150 pool screening polymerase chain reaction (O-150 PCR) technique, however, was not utilized to confirm the infectivity rate due to local technical and logistical constraints, leaving uncertainty as to whether the identified third-stage of O.V larvae during dissection were of human or animal origin [7]. This study, therefore, aims to determine blackfly infectivity and annual transmission potential in the Mont Ngafula 1 medical district using O-150 PCR after four additional annual rounds of MDA following the initial entomological survey. The study hypothesized that 16 years of ivermectin mass distribution using CDTI may have transmission interruption in black flies.

## Methods

**Study design:** a longitudinal entomological study was conducted in the district of Mont Ngafula 1 in Kinshasa, the capital city of the Democratic Republic of Congo, to determine blackfly infectivity and annual transmission potential.

**Study setting and population:** Kinshasa, the capital of the DRC, is a large city (area: 9,965 km^2^), with 24 municipalities and four administrative districts, with an estimated population of 11 million (population density: ±3,600 inhabitants/km^2^) [8]. Mont-Ngafula is located in the southern region of Kinshasa, in the hilly areas of rural Kimwenza occupied by the Lukaya River [8]. The study was conducted over 24 weeks in the medical district of Mont Ngafula 1. The description of the study site was extensively presented in the first entomological study conducted from August 2014 to July 2015 [6]. The collection point of blackflies was at the Kimwenza site (4° 27' 33'' South, 15° 17' 20'' East). The population of this study was female blackflies. Different materials and reagents were used in this study. Isopropanol, 95% ethanol, 96-Well Flipper tube Rack, 1.5-mL/1.7ml Eppendorf tubes, 50-mL conical screw-capped polypropylene tubes, petri dishes of different sizes, transfer pipette, 25-mesh sieve, and pan, (-70°C) freezer or liquid nitrogen were used for the separation of heads from the bodies of the flies.

For the isolation of DNA from pools of heads, the following materials and reagents were used: 95% ethanol, TE buffer, 10mM Tris-HCl, 1mM EDTA (pH 8.0), 10mg/ml proteinase K, 1M DTT, 1M Tris-HCl (pH 7.5), 4M NaCl, 0.5uM OVS2-biotin. (5'B-AATCTCAAAAAACGGGTACATA-3', where B= biotin), dynabeads M-280 streptavidin. Invitrogen #112-05D, beads binding buffer (100 mM Tris-HCl (pH 7.5) 100 mM NaCl), PCR water, 1- 96-well flipper tube rack, 1.5-mL/1.7ml Eppendorf tubes, disposable blue plastic homogenizer, Block heater, (-70°C) freezer and -20°C freezer, centrifuge, 96-well PCR plate, 96-well magnet, 1.5ml tube rocker, multi-channel pipet, thermocycler, 96-well PCR plate thermo sealer, pipettes, aerosol barrier tips (20ul, 200ul, 1000ul). For PCR amplification for the O-150 the following reagents and materials were used 10X PCR buffer, 2mM each dNTP, 20uM primers, taq polymerase, pipettes, aerosol barrier tips (20ul, 200ul, 1000ul), 96-well flipper tube rack, 1.5ml/15ml tubes, 96-well PCR plates, PCR plate thermo sealer, thermocycler, Vortex, microfuge and ice.

**Variables:** the study variables included the daily biting rate (DBR), the monthly biting rate (MBR), the annual biting rate (ABR), the annual transmission potential (ATP), and the Blackfly infectivity rate.

### Data resource and measurement

**Data collection tool:** two blackfly catchers were trained to collect blackflies using the Human Landing Collection (HLC) technique [9].

**Data collection:** blackfly collection and morphological identification. Blackflies were collected for five consecutive days each month, from 07: 00 to 18: 00 on the collection day. The two flycatchers worked in shifts, each hour, using individual 6ml polystyrene test tubes for collection. Each tube containing the specimens was meticulously labeled with the catching point, date, and the number of caught blackflies. At the end of the day, all tubes were transported to the bioecology laboratory of the *Ecole de Santé Publique de l´Université de* Kinshasa (School of Public Health of the University of Kinshasa) for detailed morphological examination and identification of species. In the laboratory, flies were morphologically identified based on dichotomous keys proposed by Crosskey and preserved in alcohol 80% before shipment to the molecular laboratory of the Expanded Special Program for the Elimination of Neglected Tropical Diseases (ESPEN) located in Ouagadougou, Burkina Faso for molecular analysis using O-150 PCR assay [10]. Processing of blackflies in the ESPEN Laboratory. At the ESPEN Laboratory in Ouagadougou, meticulous secondary sorting of shipped blackflies was conducted. The sorting process involved forming pools based on the daily collection of blackflies, each containing 100 to 200 blackflies. For processing, the heads and bodies of the flies within each pool were separated. This separation was achieved by freezing the flies, agitation, and sifting through a 25-mesh sieve. Only the head pools were processed. DNA extractions were performed in sets of 20 samples, each comprising 18 pools of fly heads, and 2 sham extractions to ensure the extraction process remained contamination-free. The purified DNA samples from these head pools were then tested for *O. volvulus* parasites using the O-150 PCR assay [10-12].

**Sample size:** pool screening of female black flies usually requires PCR analysis of at least 6000 flies per transmission zone according to WHO elimination criteria. However, in this study, only 3,875 female blackflies were caught and processed [10].

**Data analysis:** the data were analyzed using Excel™ and SPSS 20™ data analysis Software. O-150 PCR pool screening software was used to calculate the infectivity rate and annual transmission potential and their 95% confidence intervals.

**Ethical consideration:** the study received ethical clearance from the ethics committee of the School of Public Health of the University of Kinshasa (approval number ESP/CE/139/2019). In compliance with the ethical principles outlined in the Declaration of Helsinki, all data were collected following stringent ethical guidelines. Informed and written consent was obtained from the two flycatchers who participated as human bait in this study. The two flycatchers did not receive any financial incentives for their participation. However, they were provided with medical examinations as part of ethical and safety considerations. The two flycatchers alternated every hour to minimize risk and exposure and reduce their exposure times.

## Results

**Vector identification:** morphological examination confirmed that all specimens belonged to the *Simulium squamosum* species.

**Figure 1:**
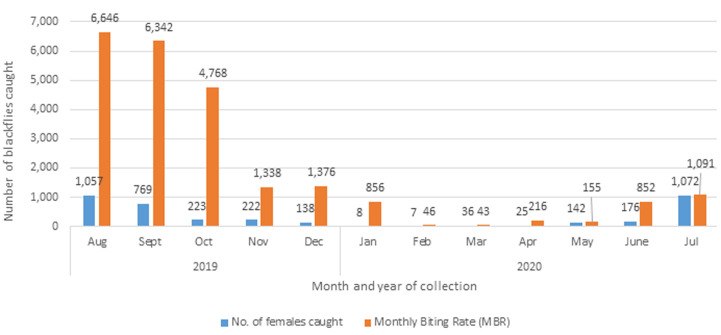
number of collected blackflies and monthly biting rate

**Number of collected blackflies and monthly biting rates:** a cumulative 3,875 female blackflies were collected from the Kimwenza study site in the medical district of Mont Ngafula 1 from August 2019 to July 2020. The DBR and MBR were 775 and 23,729, respectively. The highest capture month of blackflies was August, corresponding to the beginning of the rainy season. The decline in fly collection was observed from September to May of the following year. The biting rate was exceptionally high in August 2019. Following this period, there was a gradual decline in the biting rate, reaching its lowest in May 2020. The trend reversed with an increase in the biting rate noted from June to July 2020 during the dry season (Table 1, Figure 1).

**Table 1:** number of blackflies collected and calculated biting rates (DBR, MBR and ABR) from August 2019 to July 2020 at the Kimwenza capture sites

Parameters	2019					2020							Total
	Aug	Sept	Oct	Nov	Dec	Jan	Feb	Mar	Apr	May	June	Jul	
No of capture days	5	5	5	5	5	5	5	5	5	5	5	5	60
No of females caught	1057	769	223	222	138	8	7	36	25	142	176	1072	3875
Daily biting rate (DBR)	214	211	154	45	44	28	2	1	7	5	28	35	775
Monthly biting rate (MBR)	6646	6342	4768	1338	1376	856	46	43	216	155	852	1091	23,729
Annual biting rate (ABR)													23,729

**Infectivity rate and annual transmission potential:** the blackfly infectivity rate and annual transmission potential calculated using the O-150 PCR pool screening assay were 0.75% (95% CI: 0.48-1.13) and 254 (95% CI: 153-396), respectively.

## Discussion

This study aimed to determine blackfly infectivity rate and annual transmission potential in the Mont Ngafula 1 medical district using O-150 PCR after four additional annual rounds of MDA following an initial entomological survey from August 2014 to July 2015. The morphological examination of collected adult females revealed that they all belonged to the species *Simulium squamosum* of the *S. damnosum s.l*. complex; coinciding with results from previous surveys by Makenga in Kinshasa [6]. Henry *et al*. also identified the vectors of *O. volvulus* to belong to the *S. damnosum s.l*. complex in Kinsuka in 1984 and 1985 [13,14]. Monthly variation patterns in biting rates found in this study area align with previous observations in Kinshasa [6,15]. However, the annual biting rate of 23,729 bites per person per year is very high. The increase in blackfly numbers beginning in June and continuing in July coincides with the dry season in Kinshasa and Brazzaville, the capital cities of the DRC and the Republic of Congo, respectively. This pattern also aligns with the findings of Lapy *et al*. in Brazzaville in the Republic of Congo [15]. This pattern suggests a seasonal preference in the breeding behavior of *Simulium squamosum*, with implications for the timing of vector control interventions, if possible, and community ivermectin mass distributions. This study shows the infectivity rate and its 95% confidence interval upper bound above the WHO-recommended threshold of 0.05% [6,7].

The annual transmission potential was 10 times above the recommended value of 20 [6,7]. These results show an ongoing transmission of human onchocerciasis in the Mont Ngafula 1 medical district despite 16 years of mass drug administration using the CDTI strategy and confirm our results observed during the first entomological study [10]. Several potential driving factors could explain the ongoing transmission of onchocerciasis in Mont Ngafula 1. The first potential driving factor could be the implementation of MDA during the low transmission session. The review of MDA data from 2003 to 2019 shows that ivermectin mass campaigns in Kinshasa were implemented at the end of the fourth quarter of the year ( November-December) because of either the late arrival of Ivermectin or funds. Implementing MDA campaigns towards the end of the year instead of targeting the high transmission season is a missed opportunity and contributes to the ongoing transmission. The second driving factor is the reliability of reported treatment coverage. Despite the reporting of effective treatment coverage by the national neglected tropical program in the Democratic Republic of Congo, no post-treatment coverage surveys, as recommended by the WHO, have ever been conducted in the three districts endemic for onchocerciasis in Kinshasa because of limited funds. There is a need for the program to conduct coverage supervisory surveys during the MDA to identify areas needing mop-up and independent post-treatment coverage surveys to confirm reported treatment figures and identify potential areas of improvement [16].

The third potential driving factor could be the non-compliance of communities to treatment. Several studies have documented non-adherence to CDTI in rural and urban settings [17,18]. Programmatic or individual factors cause this non-adherence to CDTI. Programmatic factors include Community Drug Distributors (CDD) not reaching communities when people are around because of their daily activities and poor planning and implementation of MDA. On the other hand, individual issues include fear of developing serious adverse events, being not convinced to be sick and therefore not required to take Ivermectin tablets, and not being aware of mass campaigns. The non-adherence of communities and individuals to mass campaigns presents a higher risk of sustaining the ongoing transmission [17,19]. The fourth, but not the least, driving factor of the ongoing transmission is the sharing of the biological transmission zone shared by the two cities, Kinshasa and Brazzaville. Lapy *et al*. [15] showed an ongoing transmission of human onchocerciasis in the Djoué zone despite two decades of CDTI. This study had some limitations. The first limitation was the catching of blackflies from one collection site. The authors should have considered three to four collection sites in Kimwenza to have a holistic view of the onchocerciasis entomological situation in the whole of Mont Ngafula. Unfortunately, this was not possible because of very limited funds.

## Conclusion

This study showed blackfly infectivity rate and annual transmission potential above the WHO-recommended threshold. It confirmed an ongoing transmission of O.V. infection despite 16 years of annual ivermectin mass distribution using the CDTI strategy. These findings confirm the results of the first entomological studies, which used dissection, not the O-150 PCR assay. To accelerate the transmission interruption and meet the 2030 WHO road map targets, there is an urgent need for the National Onchocerciasis Program in the Democratic Republic of Congo to stop doing business as usual by changing its treatment strategies. The first recommendation to the program is to distribute ivermectin between June and July before the high transmission season. The second recommendation is to plan and conduct independent treatment coverage surveys to verify the accuracy of treatment coverage reported by community drug distributors. The third recommendation to the program is to move from annual to bi-annual ivermectin mass distribution and, finally, couple MDA with a slash and clear strategy in Mont Ngafula to reduce nuisance and accelerate the interruption and elimination of transmission.

### What is known about this topic

- Blackflies are known to transmit Onchocerca volvulus, which causes human onchocerciasis;
- The distribution of ivermectin using community-directed treatment with ivermectin strategy is a recommended World Health Organization core strategy to interrupt the transmission of human onchocerciasis when individuals and endemic communities adhere to treatments.

### What this study adds

- This study provides data on the appropriate period to implement ivermectin mass distribution using community-directed treatment with ivermectin in Kinshasa;
- This study provides evidence of the ongoing transmission of onchocerciasis in Kinshasa despite 16 years of annual mass distribution of ivermectin using CDTI strategy.

## References

[1] Hadermann A, Jada SR, Sebit WJ, Deng T, Bol YY, Siewe Fodjo JN (2023). Onchocerciasis-associated epilepsy: an explorative case-control study with viral metagenomic analyses on *Onchocerca volvulus*. F1000Res.

[2] Hotterbeekx A, Namale Ssonko V, Oyet W, Lakwo T, Idro R (2019). Neurological manifestations in Onchocerca volvulus infection: A review. Brain Res Bull.

[3] Organisation mondiale de la Santé (OMS), World Health Organization (WHO) (2022). Elimination of human onchocerciasis: progress report, 2021-Élimination de l’onchocercose humaine: rapport de situation, 2021. Wkly Epidemiol Rec Relev épidémiologique Hebd.

[4] World Health Organization (WHO) (2023). Weekly Epidemiological Record (WER). WHO.

[5] Makenga Bof JC, Ntumba Tshitoka F, Muteba D, Mansiangi P, Coppieters Y (2019). Review of the National Program for Onchocerciasis Control in the Democratic Republic of the Congo. Trop Med Infect Dis.

[6] Makenga Bof JC, Mukendi DM, Molala R, Ilunga-ilunga F, Coppieters Y (2017). Analysis of the transmission level of onchocerciasis in a health area in Kinshasa, Democratic Republic of Congo (DRC). Journal of Entomology and Zoology Studies.

[7] World Health Organization (WHO) (2016). Guidelines for stopping mass drug administration and verifying elimination of human onchocerciasis: Criteria and Procedures. World Heal Organ WHO/HTM/NTD/PCT Geneva, Switz.

[8] Ministère de la Santé Publique Secrétariat Général RDC (2016). Plan Stratégique de Lutte contre les Maladies Tropicales Négligées à Chimiothérapie Préventive 2016-2020. Ministère de la Santé Publique, Secrétariat Général, RDC, Kinshasa.

[9] Walsh JF, Davies JB, Le Berre R, Garms R (1978). Standardization of criteria for assessing the effect of simulium control in onchocerciasis control programmes. Trans R Soc Trop Med Hyg.

[10] World Health Organization (WHO) (2023). Entomological manual for onchocerciasis elimination programmes. World Health Organization, Geneva.

[11] Katholi CR, Toé L, Merriweather A, Unnasch TR (1995). Determining the prevalence of Onchocerca volvulus infection in vector populations by polymerase chain reaction screening of pools of black flies. J Infect Dis.

[12] Yamèogo L, Toè L, Hougard JM, Boatin BA, Unnasch TR (1999). Pool screen polymerase chain reaction for estimating the prevalence of Onchocerca volvulus infection in Simulium damnosum sensu lato: results of a field trial in an area subject to successful vector control. Am J Trop Med Hyg.

[13] Henry MC, Janssens PG, De Boeck M (1984). Observations récentes sur la transmission de l'onchocercose à Kinsuka, Kinshasa, Zaïre. Ann Soc Belg Med Trop.

[14] Henry M-C, Meredith SEO (1990). The onchocerciasis focus at Kinsuka/Kinshasa (Republic of Zaire) in 1985: I. Entomological aspect. Ann Trop Med Parasitol.

[15] Lapy Mathah SE, Lenga A, Bitsindou P, Missamou F (2022). Evaluation of some parameters involved in onchocerciasis transmission in the DJOUE fireplace in Brazzaville 2022. Journal of Entomology and Zoology Studies.

[16] World Health Organization (WHO) (2019). Preventive chemotherapy: tools for improving the quality of reported data and information a field manual for implementation. WHO.

[17] Senyonjo L, Oye J, Bakajika D, Biholong B, Tekle A, Boakye D (2016). Factors Associated with Ivermectin Non-Compliance and Its Potential Role in Sustaining Onchocerca volvulus Transmission in the West Region of Cameroon. PLoS Negl Trop Dis.

[18] Kamga GR, Dissak-Delon FN, Nana-Djeunga HC, Biholong BD, Ghogomu SM, Souopgui J (2018). Audit of the community-directed treatment with ivermectin (CDTI) for onchocerciasis and factors associated with adherence in three regions of Cameroon. Parasit Vectors.

[19] Bakajika D, Senyonjo L, Enyong P, Oye J, Biholong B, Elhassan E (2018). On-going transmission of human onchocerciasis in the Massangam health district in the West Region of Cameroon: Better understanding transmission dynamics to inform changes in programmatic interventions. PLoS Negl Trop Dis.

